# Avian Orthoreovirus in China: Molecular Evolution, Transmission Ecology, Immune Modulation, and Integrated Control in the Genomic Era

**DOI:** 10.3390/v18070728

**Published:** 2026-06-30

**Authors:** Lijuan Yin, Peier Huang, Yanhua Xu, Ouyang Peng, Kensi Zhu, Ermin Xie, Shenghua Yang, Jin Liu, Xuesong Li, Zhuanqiang Yan, Jianping Qin, Wencheng Lin

**Affiliations:** 1 Wen’s Foodstuffs Group Co., Ltd., Yunfu 527400, China; wsyinlj@163.com (L.Y.);; 2 Yunfu Branch, Guangdong Laboratory for Lingnan Modern Agriculture, Yunfu 527400, China; 3 College of Animal Science, South China Agricultural University, Guangzhou 510642, China; pearlhuang1007@163.com (P.H.);

**Keywords:** avian orthoreovirus, reassortment, vaccine escape, σC protein, immunosuppression, evolutionary ecology, precision control, China

## Abstract

Avian orthoreovirus (ARV) has re-emerged as one of the most important viral pathogens affecting modern poultry production worldwide. In China, the epidemiological landscape of ARV has undergone a substantial transformation over the past decade, characterized by increasing genotypic diversity, frequent genome reassortment, an expanding host range, and recurrent vaccine-breakthrough outbreaks. Growing evidence indicates that contemporary ARV populations evolve within a dynamic multispecies transmission network shaped by intensive poultry production, host adaptation, and vaccine-associated selective pressures. Recent molecular studies have revealed extensive genetic heterogeneity among circulating strains and highlighted the limitations of conventional σC-based classification systems for accurately describing viral evolution, pathogenicity, and antigenic diversity. Whole-genome analyses further demonstrate that reassortment among chicken-origin, duck-origin, and goose-origin orthoreoviruses plays a pivotal role in generating novel viral variants with altered biological properties. In parallel, accumulating evidence suggests that ARV exerts broad immunomodulatory effects through the disruption of innate antiviral signaling, impairment of lymphoid organ function, interference with vaccine responsiveness, and the enhancement of susceptibility to secondary infections. These findings indicate that ARV should be regarded not only as an arthrotropic pathogen but also as an important immunopathological agent influencing flock health and productivity. This review summarizes current knowledge of ARV in China, with an emphasis on molecular epidemiology, genomic evolution, reassortment mechanisms, transmission ecology, immune interference, vaccine escape, and integrated prevention strategies. Particular attention is given to the increasing importance of whole-genome surveillance, phylodynamic analysis, and multispecies epidemiological monitoring for understanding contemporary ARV evolution. Future perspectives involving structural vaccinology, precision immunization, metagenomics-assisted surveillance, and predictive evolutionary modeling are also discussed. Collectively, sustainable ARV control will likely require genome-informed and adaptive prevention frameworks integrating virology, immunology, epidemiology, and precision poultry management.

## 1. Introduction

Avian orthoreovirus (ARV), a non-enveloped double-stranded RNA virus of the genus *Orthoreovirus* within the family *Spinareoviridae* and order *Reovirales*, has emerged as an increasingly important pathogen in modern poultry production systems [1]. Since its initial isolation from chickens with chronic respiratory disease in 1954 [2], ARV has gradually shifted from a relatively overlooked avian virus into a globally distributed pathogen associated with substantial economic losses in the commercial poultry industry. ARV infects a broad range of avian hosts, including chickens, turkeys, ducks, geese, and quails [2,3,4]. Among susceptible species, broiler chickens and turkeys are particularly vulnerable to viral arthritis and tenosynovitis, which are regarded as the most economically significant manifestations of ARV infection worldwide [5].

ARV infection causes a diverse range of clinical and pathological outcomes, including viral arthritis/tenosynovitis, enteric disease, runting-stunting syndrome, malabsorption syndrome, myocarditis, hepatitis, immunosuppression, and impaired production performance [6]. In commercial broiler production, the disease is most commonly recognized by lameness, swelling of the hock joints, tendon rupture, poor weight gain, reduced feed conversion efficiency, and increased carcass condemnation at processing [7]. Severe outbreaks may produce morbidity exceeding 15%, causing substantial economic losses through impaired flock uniformity, increased culling rates, greater antimicrobial use, and processing losses [8,9].

Historically, ARV control relied primarily on live attenuated and inactivated vaccines derived from classical vaccine strains such as S1133, 1733, and 2408 [1]. For several decades, these vaccines provided effective protection against circulating field strains and substantially reduced the incidence of clinical viral arthritis in commercial poultry operations. However, beginning around 2011, vaccine-breakthrough ARV variants emerged in North America, Europe, and Asia, causing severe disease outbreaks even in vaccinated flocks [10,11,12,13]. Since then, China has become an important region for ARV genetic diversification, variant emergence, and epidemic dissemination.

The rapid intensification of poultry production systems in China has created ecological and management conditions that are highly conducive to ARV transmission and evolution [14]. Factors such as high stocking density, vertically integrated production systems, mixed-species poultry farming, long-distance transportation of live birds, suboptimal biosecurity implementation, and sustained vaccine-associated immune selection may collectively facilitate viral transmission, host adaptation, mutation accumulation, and genomic reassortment [15]. Compared with traditional farming systems, industrialized poultry production creates more continuous and large-scale opportunities for viral amplification and co-infection, thereby shaping the evolutionary dynamics of circulating ARV populations [16].

A major driver of ARV diversification is its segmented genome organization. The ARV genome consists of ten double-stranded RNA segments, which are grouped into three size classes: large (L1–L3), medium (M1–M3), and small (S1–S4) segments, encoding at least twelve structural and non-structural proteins [17,18]. Such a segmented genome provides an intrinsic genetic basis for rapid diversification through both point mutations and genomic reassortment during co-infection [19]. Recent whole-genome analyses of 19 representative ARV isolates collected from Chinese commercial broiler farms between 2016 and 2021 demonstrated that the σC-encoding segment clustered into six distinct phylogenetic lineages, whereas the remaining genomic segments formed only two to four lineages, suggesting marked heterogeneity in evolutionary rates among different genome segments [12]. These findings suggest that reliance solely on σC-based classification may underrepresent the virus’s genomic complexity and evolutionary plasticity. Among ARV proteins, the σC protein encoded by the S1 segment exhibits the greatest genetic variability and functions as both the principal cell attachment protein and a major target of viral neutralizing antibodies [20,21,22]. Accordingly, σC-based phylogenetic analysis has long been widely used for ARV genotyping and epidemiological investigation [23,24,25]. Nevertheless, accumulating evidence suggests that σC-based classification alone is insufficient to fully explain differences in pathogenicity, host adaptation, tissue tropism, transmission fitness, immune escape, and epidemiological success among contemporary ARV strains [9,26,27]. Increasingly, whole-genome sequencing studies have revealed frequent and complex reassortment among chicken-origin ARVs, novel duck reoviruses (NDRV), and goose-origin reoviruses (GRV), highlighting the limitations of single-gene-based evolutionary frameworks [28,29]. A recent comprehensive phylogenetic investigation of duck and goose reoviruses circulating in China further demonstrated multiple reassortment events involving duck reovirus, avian reovirus, and Muscovy duck reovirus lineages [30]. The emergence of these reassortant viruses suggests that multispecies poultry production and interspecies transmission interfaces may serve as important reservoirs for ARV genetic diversification and evolutionary innovation.

Another important characteristic of contemporary ARV infection is its capacity to modulate host immune responses. Beyond causing direct tissue injury, ARV can disrupt both innate and adaptive immune responses, thereby increasing host susceptibility to secondary bacterial and viral pathogens. ARV employs multiple immune-evasion mechanisms; notably, the σA protein inhibits type I interferon production [31], while the p17 protein interacts with IFI16 and PQBP1 to suppress inflammatory signaling [32,33]. Co-infections involving chicken anemia virus (CAV), fowl adenovirus serotype 4 (FAdV-4), *Mycoplasma synoviae* (MS), infectious bursal disease virus (IBDV), *Staphylococcus aureus*, and pathogenic *Escherichia coli* can aggravate clinical disease, increase flock-level losses, and potentially interfere with vaccine performance [34,35,36,37,38]. Accordingly, ARV is increasingly recognized as a systemic immunomodulatory pathogen, with effects that extend beyond musculoskeletal disease to influence polymicrobial interactions, vaccine responsiveness, and overall flock health.

Given the rapidly evolving epidemiological and genetic landscape of ARV, a comprehensive reassessment of its evolutionary biology, transmission ecology, immune interference, and prevention strategies in China is urgently needed. This review synthesizes recent advances in ARV research, with a particular emphasis on the context of the Chinese intensive poultry industry, covering molecular epidemiology, genomic evolution, transmission dynamics, immune modulation, and integrated control strategies. Special attention is devoted to whole-genome surveillance, reassortment analysis, multispecies transmission interfaces, and vaccine-driven evolutionary processes, all of which are critical for understanding the diversification and persistence of contemporary ARV populations. Finally, future perspectives on genomic surveillance, vaccine innovation, predictive epidemiology, and precision prevention are discussed within the broader context of intensive poultry production, multispecies farming systems, and viral evolution.

## 2. Molecular Epidemiology of ARV in China

Over the past two decades, the epidemiological profile of ARV infection in China has undergone a substantial transformation. Earlier outbreaks were typically sporadic and were largely associated with classical viral arthritis, which could be relatively well controlled by conventional vaccination programs. Since approximately 2010, however, the epidemiological situation has shifted markedly toward persistent vaccine-breakthrough outbreaks, sustained breeder-flock-associated transmission, and rapid expansion of genetically heterogeneous viral populations [12,25]. These observations collectively indicate that ARV has transitioned from a relatively localized cause of viral arthritis to a widely distributed and evolutionarily dynamic pathogen characterized by extensive genetic diversification, recurrent vaccine-breakthrough infections, and increasing epidemiological complexity across Chinese intensive poultry production systems.

The rapid intensification of poultry production in China has likely created ecological and management conditions that are conducive to ARV dissemination and adaptive evolution. High stocking density, vertically integrated production systems, extensive live-bird movement, mixed-species farming practices, suboptimal biosecurity implementation, and sustained vaccine-associated immune selection may collectively promote viral transmission, reassortment, and host adaptation [14]. Accordingly, the molecular epidemiology of contemporary ARV populations is increasingly characterized by dynamic lineage turnover, reassortment-driven diversification, and occasional interspecies transmission, suggesting a continuously evolving viral population structure rather than the long-term persistence of genetically stable lineages.

### 2.1. China as a Hotspot for ARV Genotypic Diversification

**Figure 1:**
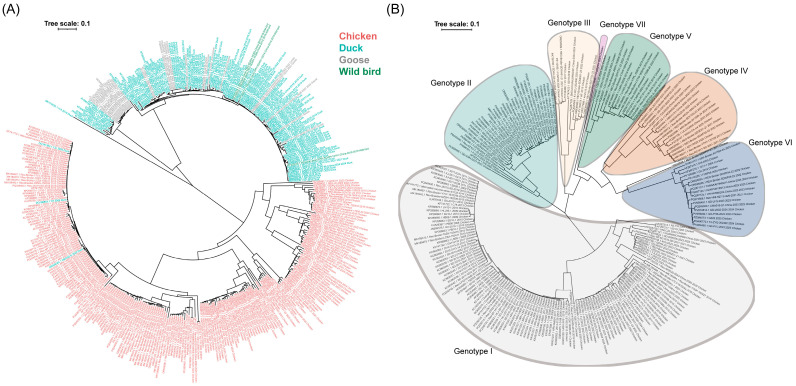
Phylogenetic relationships and genotypic classification of avian orthoreoviruses (ARVs) circulating in China based on *σC gene* sequences. (**A**) Neighbor-Joining phylogenetic tree constructed using all available *σC gene* sequences of chicken-, duck-, goose-, and wild-bird-origin ARVs reported in China and deposited in GenBank (accessed in 2025). Chicken-origin ARVs are indicated in red, duck-origin ARVs in blue, goose-origin ARVs in gray, and wild-bird-origin ARVs in green. Three duck-origin isolates clustered within the chicken-origin ARV lineage, suggesting potential interspecies transmission between waterfowl and chickens. (**B**) Neighbor-Joining phylogenetic tree constructed using *σC gene* sequences from Chinese chicken-origin ARV isolates only. Seven genotypic clusters (GI-GVII) were identified based on phylogenetic topology and sequence divergence, demonstrating substantial genetic diversity among circulating chicken-origin ARVs in China. A putative genotype VII (GVII) lineage is highlighted, representing a recently recognized and genetically distinct phylogenetic cluster that requires further epidemiological, antigenic, and biological characterization.

To systematically investigate the evolutionary diversity of ARVs circulating in China, we conducted a comprehensive phylogenetic analysis based on all currently available *σC gene* sequences of poultry- and wild-bird-derived avian orthoreoviruses deposited in GenBank. The resulting Neighbor-Joining phylogeny revealed clear host-associated clustering, with most chicken-, duck-, and goose-origin ARVs forming distinct phylogenetic lineages (Figure 1A). Notably, several duck-origin isolates were embedded within chicken-origin ARV lineages, providing phylogenetic evidence suggestive of occasional cross-species transmission between waterfowl and chickens. These findings further support the emerging view that waterfowl-associated orthoreoviruses are not evolutionarily isolated viral populations, but rather constitute part of a broader multispecies transmission network characterized by intermittent host spillover and potential genomic exchange [30].

Focused phylogenetic analysis of chicken-origin ARVs identified seven genotypic clusters (GI-GVII) according to phylogenetic topology and sequence divergence (Figure 1B). This classification expands upon the previously proposed six-genotype framework and further underscores the ongoing diversification of ARV populations circulating in China [25,39]. Notably, among the seven identified genotypes, a putative genotype VII (GVII) lineage was recognized. This lineage is represented by a limited number of recently reported isolates that form a distinct and well-supported phylogenetic branch separate from previously recognized genotypes. The lineage also exhibited substantial σC sequence divergence from representative strains of GI-GVI and formed an independent phylogenetic cluster, supporting its provisional designation as a separate genotype. Preliminary sequence comparisons further indicate considerable divergence from classical vaccine strains. However, the epidemiological distribution, antigenic characteristics, pathogenicity, and evolutionary origins of GVII remain poorly understood due to the limited number of available isolates. Further genomic, antigenic, and biological characterization will be required to determine its epidemiological significance and potential implications for vaccine effectiveness. Despite these uncertainties, the identification of GVII further highlights the continuing diversification of ARV populations circulating in China.

Current surveillance data indicate that all seven recognized genotypic groups (GI-GVII) have been detected in China, with several lineages showing considerable intra-genotypic variability [25,40]. Surveillance conducted during 2019–2020 identified GI and GII as the predominant genotypes in commercial broiler populations [40]. In contrast, surveillance spanning 2010–2024 reported a marked increase in GVI-related strains, which constituted the largest genotype cluster within that dataset [25]. Interpretation of genotype prevalence should nevertheless be approached cautiously because apparent dominance may be influenced by differences in sampling period, geographic coverage, host population, production type, and surveillance intensity. Collectively, these findings suggest that the historical predominance of GI strains has gradually shifted toward a genetically heterogeneous ARV population characterized by the co-circulation of multiple lineages, periodic shifts in genotype prevalence, and substantial regional variation.

The observed shifts in genotype prevalence indicate that contemporary ARV populations are unlikely to remain under long-term evolutionary equilibrium. Instead, periodic lineage expansion and replacement appear to be persistent features of ARV population dynamics. Similar genotype-transition patterns have been reported for other rapidly evolving avian RNA viruses, including avian influenza virus [41] and infectious bronchitis virus [42], suggesting that immune-mediated ecological selection pressures may be a recurrent driver of viral population turnover in intensive poultry production systems.

**Figure 2:**
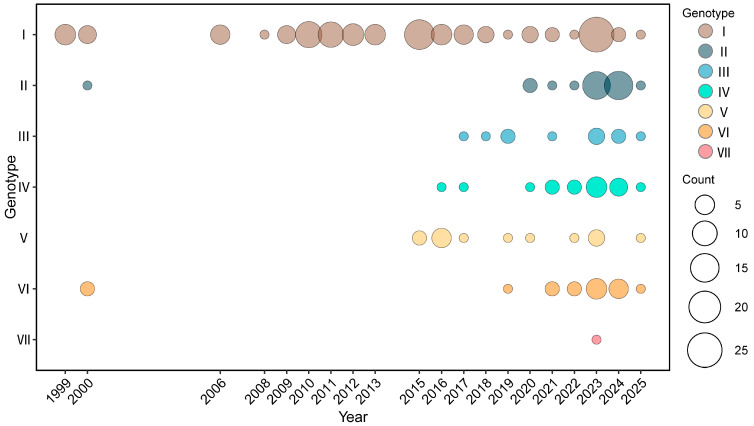
Temporal distribution and genotype replacement patterns of chicken-origin avian orthoreoviruses in China from 1999 to 2025. The bubble plot illustrates the temporal prevalence of seven avian orthoreovirus (ARV) genotypes across different years. The vertical axis represents genotypes GI-GVII, whereas the horizontal axis indicates the year of virus isolation. Each circle represents a genotype identified in a specific year, and circle size is proportional to the number of isolates. Distinct colors differentiate the seven genotypes. Genotype GI predominated during the early surveillance period (1999–2013), whereas multiple emerging genotypes (GII-GVII) became increasingly prevalent after 2015. The simultaneous circulation of multiple genotypes during 2015–2025 highlights the increasing epidemiological complexity and dynamic lineage replacement of contemporary ARV populations in China. Different color of circles indicate the genotypes of ARV.

The increasing genetic distance between contemporary field isolates and historical reference strains further highlights the continued diversification of ARV populations in China. For example, strain FJ202311 formed an independent phylogenetic branch and showed remarkably low σC amino acid identity showed remarkably low σC amino acid identity with previously recognized genotypes, suggesting that additional unsampled or currently unrecognized genetic diversity may exist outside existing classification frameworks [39]. The temporal distribution of genotypes (Figure 2) reveals a gradual shift from early GI predominance to the co-circulation of multiple non-vaccine genotypes after approximately 2015. Importantly, available evidence does not support a simple pattern of nationwide replacement from one dominant lineage to another. Rather, genotype composition appears to fluctuate across surveillance periods and geographic regions, reflecting the dynamic and regionally heterogeneous nature of ARV evolution in China. The concurrent circulation of multiple genetically divergent lineages within the same production systems likely increases opportunities for co-infection and genomic reassortment, thereby futher promoting viral diversification under field conditions.

### 2.2. Vaccine-Field Strain Divergence and Potential Immune Escape

Commercial ARV vaccines currently used in China are largely derived from classical genotype I (GI) reference strains, including S1133, 1733, and 2408. In contrast, most contemporary field isolates belong to heterologous genotypes (GII-GVII) and frequently exhibited marked σC sequence divergence from vaccine strains [39,43,44]. Importantly, genetic diversity and antigenic diversity should not be regarded as synonymous concepts. Genetic diversity refers to variation at the nucleotide or amino acid level across viral genomes, whereas antigenic diversity reflects differences in immune recognition, particularly by neutralizing antibodies. Although genetic divergence may contribute to antigenic variation, the relationship between these two properties is not necessarily linear. Genetically distinct viruses may remain antigenically related, whereas limited amino acid substitutions occurring within critical neutralizing epitopes can produce substantial antigenic changes. Consequently, phylogenetic distance alone cannot reliably predict vaccine cross-protection. Nevertheless, increasing genetic divergence between vaccine strains and circulating field isolates may elevate the likelihood of antigenic mismatch and contribute to vaccine-breakthrough outbreaks. Therefore, genomic surveillance should be complemented by cross-neutralization studies and epitope characterization to better assess the antigenic relevance of emerging ARV variants.

Several recent studies demonstrated that certain field strains share only slightly more than 50% σC nucleotide identity with classical GI vaccine strains, suggesting pronounced antigenic divergence and potential reductions in cross-protective immunity [25,45]. Structural visualization of the σC protein further revealed that amino acid substitutions are predominantly concentrated in the globular C-terminal domain, which is involved in receptor binding, thereby providing a plausible structural basis for antigenic variation and potential immune escape [46].

Importantly, antigenic divergence is not restricted to comparisons among distinct genotypes. Even within GI, several contemporary field isolates have formed vaccine-distinct sublineages that exhibit reduced cross-neutralization against antisera raised to classical GI vaccine strains [43,47]. These findings reinforce the view that σC-based genotype classification alone is insufficient for accurately predicting antigenic relatedness and vaccine-mediated cross-protection. Although σC remains an important epidemiological marker, exclusive reliance on partial σC phylogeny may oversimplify the antigenic and evolutionary complexity of contemporary ARV populations. Increasing evidence indicates that effective vaccine matching will require an integrated framework that combines genomic surveillance, cross-neutralization assays, structural epitope mapping, and whole-genome evolutionary analysis rather than conventional genotype assignment alone [9,27,43].

Suboptimal or incomplete immunity may further accelerate ARV evolution under field conditions. When vaccine-induced immune pressure fails to fully suppress viral replication, partially resistant variants may retain sufficient replication fitness while gaining a selective advantage through immune escape [24]. Such evolutionary dynamics are consistent with theoretical models of pathogen adaptation under partial immune pressure, in which viral populations capable of replicating in incompletely protected hosts are provided with opportunities for persistent selection, and adaptive diversification. Similar processes have been implicated in the evolutionary trajectories of several rapidly evolving RNA viruses under sustained vaccine-induced or naturally acquired population immunity.

### 2.3. Regional Heterogeneity, Host Expansion, and Co-Infection Ecology

ARV circulation in China remains predominantly concentrated in major poultry-producing regions, including Shandong, Jiangsu, Fujian, Guangdong, and Guangxi Provinces, where dense breeder networks, hatchery systems, and continuous live-bird movement may facilitate sustained viral circulation and regional dissemination [25,40,48]. Clinical outbreaks occur most frequently in broilers 15–45 days of age, corresponding approximately to the progressive decline of maternally derived antibodies [5,49]. This age-associated susceptibility window may promote efficient viral amplification and onward transmission under intensive production conditions.

In parallel with increasing genetic diversity, the host spectrum of avian orthoreoviruses has broadened substantially over the past two decades. Novel duck reovirus and goose-origin reovirus are now widely distributed in waterfowl-producing regions of China and are associated with severe systemic disease, including hemorrhagic hepatitis, splenic necrosis, arthritis, and elevated mortality [29,30,50,51].

The increasing ecological overlap between chicken and waterfowl production systems, particularly in southern China where integrated duck-goose-chicken farming remains common, may provide interfaces for viral exchange and reassortment. Although direct evidence for sustained interspecies transmission remains limited, phylogenetic and experimental studies increasingly support the hypothesis that waterfowl-associated orthoreoviruses contribute to the broader evolutionary gene pool of poultry-associated orthoreoviruses [29,52]. Notably, experimental infection studies demonstrated that chickens are susceptible to circulating duck-origin reoviruses, thereby providing experimental support for potential cross-species transmission at the waterfowl–chicken interface [30].

Co-infections further complicate the epidemiological consequences of ARV infection. Field and experimental studies have demonstrated that ARV co-infection with *Staphylococcus aureus* or fowl adenovirus serotype 4 can result in more severe tenosynovitis, systemic inflammatory lesions, and higher mortality compared to mono-infections [34,35]. Recent virome-based investigations further suggest that the pathogenesis of enteric and malabsorption syndromes in broilers is unlikely to be attributable to a single pathogen [37]. Instead, multiple enteric viruses may act synergistically to exacerbate intestinal dysfunction and production losses [53]. These findings support the concept that ARV frequently participates in complex polymicrobial disease networks that contribute to the clinical and economic burden of poultry production. The biological basis underlying these synergistic disease outcomes is likely linked to ARV-associated immune dysregulation and impaired host defense mechanisms [31,54]. The mechanistic basis of these immunomodulatory effects is discussed in detail in Section 5.

Taken together, the contemporary epidemiology of ARV in China is increasingly characterized by genetic diversification, ecological expansion, and polymicrobial interactions. These interconnected processes collectively indicate a shift from a relatively predictable endemic infection pattern toward a dynamic multispecies evolutionary system shaped by intensive poultry production and sustained immune selection.

## 3. Genomic Evolution of Avian Orthoreovirus

The evolutionary plasticity of ARV is fundamentally linked to its segmented double-stranded RNA genome [19]. Unlike viruses whose evolution is driven predominantly by the gradual accumulation of point mutations, contemporary ARV populations appear to diversify through the combined influence of mutation, segment reassortment, host-associated ecological pressures, and vaccine-mediated immune selection [28]. Within the highly interconnected production systems characteristic of modern poultry farming in China, these evolutionary mechanisms have likely contributed to the emergence of genetically and antigenically distinct variants, altered pathogenic phenotypes, and expanded host adaptability. Current evidence supports the view that ARV evolution is a dynamic population-level process characterized by continual genomic reshuffling, lineage turnover, and episodic reassortment rather than the simple linear accumulation of genetic changes.

### 3.1. The Segmented Genome as a Driver of Evolutionary Flexibility

ARV contains ten double-stranded RNA genome segments, which are grouped into large (L), medium (M), and small (S) size classes. Collectively, these segments encode at least twelve structural and non-structural proteins, including σ proteins (σA, σB, σC, σNS), P proteins (p10, p17), μ proteins (μA, μB, μNS), and λ proteins (λA, λB, λC) [55]. This segmented genomic architecture provides an intrinsic mechanism for rapid genetic diversification, enabling individual genome segments to evolve semi-independently and to be exchanged during co-infection events [19]. As a result, progeny virions may inherit novel combinations of segments derived from different parental strains, generating reassortant viruses with unique genomic constellations [28].

In parallel with reassortment, the viral RNA-dependent RNA polymerase continually generates replication-associated mutations. Because the polymerase lacks proofreading activity, replication errors can progressively accumulate, contributing to ongoing genetic diversification [19]. The interaction between mutation-driven diversification and reassortment-driven genome restructuring enables ARV populations to access a much broader evolutionary landscape than would be achievable through point mutation alone [28].

Although the σC-encoding S1 segment remains the most extensively investigated molecular marker, recent whole-genome studies increasingly demonstrate that evolutionary processes shaping ARV emergence involve genome-wide changes across multiple segments rather than σC variation alone. Mutations involving λA, μB, σA, σNS, and λC proteins have been associated with altered replication efficiency, tissue dissemination, host adaptation, and the modulation of innate immune responses [56,57,58,59]. In particular, σA has emerged as an important interferon-antagonistic protein that can suppress host antiviral signaling through the sequestration of double-stranded RNA [31,60]. Collectively, these observations indicate that ARV evolution cannot be adequately interpreted through σC variability alone but should instead be understood as a genome-wide adaptive process involving functional changes across multiple structural and non-structural proteins [27].

### 3.2. Point Mutation, Antigenic Drift, and Structural Adaptation

Accumulation of mutations within the σC protein remains one of the major mechanisms driving antigenic diversification in ARV populations. As the outer-capsid attachment protein, σC mediates host–cell recognition and represents a major target of virus-neutralizing antibodies [46,61]. Among ARV structural proteins, both σB and σC are considered key determinants of viral antigenicity [62,63]. Structural studies further demonstrated that the globular head domain of σC contains multiple surface-exposed regions that can accommodate substantial amino acid variation without completely disrupting receptor-binding function [21,46]. This structural plasticity likely contributes to antigenic drift and potential immune escape. Amino acid substitutions occurring within exposed loop regions may alter epitope accessibility or antibody-binding affinity while preserving sufficient structural stability for continued viral infectivity [15]. Consequently, ARV can accumulate antigenically significant mutations without necessarily compromising replication fitness. This balance between immune escape and preservation of biological fitness may help explains the persistence and epidemiological success of several contemporary ARV lineages circulating in China.

Field isolates from China provide supporting evidence for this evolutionary process. Recent strains exhibited numerous amino acid substitutions within σC relative to classical GI vaccine strains, particularly in putative epitope-associated regions [64]. A recent characterization of goose-origin strain SD0407 identified ten unique amino acid substitutions within σC, including residue D250 located within the DE-loop receptor-binding region. Molecular docking analysis further suggested that σC may interact with the conserved AnxA2-S100A10 heterotetrameric receptor complex, providing a possible structural explanation for broad receptor compatibility and host adaptability across avian species [65]. Codon-based evolutionary analyses further identified multiple positively selected residues within the *σC gene*, supporting the presence of on-going immune-mediated selection under field conditions [28]. Nevertheless, attributing antigenic evolution exclusively to σC variation likely oversimplifies the biology of ARV immune adaptation.

Increasing evidence indicates that adaptive mutations in non-σC proteins may substantially influence viral fitness, tissue tropism, replication efficiency, and immune evasion, highlighting the polygenic basis of ARV evolution. Alterations in μB may influence viral entry and transcriptase activation [66], whereas σA and σNS have been implicated in interferon antagonism and viral factory organization [59,67]. Notably, σA possesses strong double-stranded RNA-binding activity capable of sequestering viral replication intermediates and limiting the activation of intracellular RNA-sensing pathways, thereby suppressing type I interferon induction during early infection [31,60]. Collectively, these findings support the view that immune escape in ARV is more appropriately interpreted as a polygenic adaptive process involving multiple structural and non-structural proteins, rather than a phenomenon driven solely by σC diversification.

### 3.3. Reassortment and the Emergence of Novel Genotypes

Compared with point mutation, reassortment can generate abrupt and extensive genetic change through the exchange of complete genome segments between genetically distinct viruses. This mechanism is particularly relevant for ARV because multiple genotypes frequently co-circulate within the same production environments, creating opportunities for co-infection and segment exchange.

Whole-genome analyses of Chinese ARV isolates demonstrated extensive phylogenetic incongruence among genomic segments, indicating that reassortment is a frequent feature of ARV evolution under field conditions [68,69]. Reassortment analyses of representative Chinese ARV isolates have further identified segment exchange events involving the L3, M1, and S1 segments [12,70], providing molecular evidence for segment exchange among circulating strains. Several contemporary isolates possessed genomic constellations that could not be readily explained through simple vertical lineage evolution, strongly suggesting repeated reassortment among divergent ARV populations [28].

Importantly, reassortment is not restricted to chicken-origin ARVs. Phylogenetic analyses of duck reoviruses isolated from ducks and geese in China revealed a complex evolutionary landscape involving reassortment among duck reovirus, avian reovirus, and Muscovy duck reovirus [30]. Certain novel duck-origin reoviruses acquired genomic segments closely related to chicken-origin strains, supporting the occurrence of natural interspecies segment exchange. Some reassortant viruses also exhibit broadened adaptability and markedly increased pathogenicity in experimentally infected chickens [71].

Although the ecological stability and epidemiological frequency of these reassortment events remain incompletely understood, their evolutionary significance is likely profound. Reassortment provides a mechanism through which ARV populations can rapidly acquire new combinations of virulence-associated, host-adaptive, and immune-evasive genetic determinants, which would likely require considerably longer to emerge through point mutation alone [28]. The structure of modern poultry production systems in China likely further amplifies reassortment opportunities. High stocking density, mixed-species farming, overlapping poultry supply chains, and extensive live-bird movement collectively facilitate repeated co-infection events. Consequently, many contemporary ARV isolates may be regarded as dynamic genomic mosaics generated through continual reassortment among co-circulating viral populations.

Nevertheless, the biological consequences of most reassortment events remain incompletely understood. Although reassortment has the theoretical potential to generate viruses with altered virulence, host range, tissue tropism, transmission efficiency, or immune-evasion capacity, only a limited number of reassortant ARVs have undergone detailed phenotypic characterization. In many cases, reassortment is identified solely through genomic analyses, and the corresponding effects on viral fitness remain unknown. Moreover, not all reassortment events are necessarily adaptive, as some may be selectively neutral or transient within field populations. Consequently, caution should be exercised when inferring biological significance solely from genomic evidence. Future studies integrating whole-genome sequencing, reverse genetics, experimental infection models, and transmission studies will be essential for establishing robust genotype-phenotype relationships in reassortant ARVs.

### 3.4. Phylodynamics and Vaccine-Associated Evolutionary Selection

Phylodynamic analyses integrating genomic, temporal, and epidemiological information have provided valuable insights into the evolutionary dynamics of ARV populations in China. Available data suggest that lineage diversification may have accelerated during the period of rapid expansion of intensive poultry production and widespread vaccine application [15,25]. However, the relative contributions of vaccination, host density, management practices, and other ecological drivers remain difficult to disentangle and require further investigation.

Longitudinal surveillance has revealed repeated genotype turnover and increasing co-circulation of multiple non-vaccine lineages after approximately 2015 (Figure 2). Rather than exhibiting nationwide replacement by a single dominant genotype, ARV populations in China appear to undergo recurrent cycles of lineage expansion, coexistence, and diversification. Similar phylodynamic patterns have been described in other avian RNA viruses exposed to sustained immunological and ecological selection pressures.

Overall, current evidence indicates that contemporary ARV evolution is driven by the combined effects of point mutation, reassortment, host adaptation, and immune-mediated selection. Importantly, these processes operate across the entire viral genome rather than within the *σC gene* alone, emphasizing the necessity of genome-wide surveillance frameworks for understanding ARV emergence, vaccine escape, and long-term epidemiological dynamics. The evolutionary diversification of contemporary ARV populations has been accompanied by considerable variation in clinical presentation, tissue tropism, and pathological outcomes among different avian hosts.

## 4. Horizontal and Vertical Transmission: Viral Circulation and Persistence

The long-term persistence of ARV within commercial poultry systems depends on the continuous interplay between horizontal and vertical transmission. Rather than representing independent epidemiological routes, these pathways form an interconnected transmission network that enables sustained viral maintenance across successive production stages. Consequently, intervention strategies targeting only broiler flocks or breeder populations are unlikely to achieve durable suppression of viral circulation.

Recent epidemiological studies increasingly suggest that ARV persistence is maintained through a highly interconnected transmission network involving breeder flocks, hatcheries, commercial farms, environmental reservoirs, and subclinically infected birds. These epidemiological links enable continuous viral circulation across multiple production stages and contribute to recurrent outbreaks despite routine vaccination and biosecurity implementation. From a systems perspective, ARV transmission should be viewed as a dynamic production-chain process in which vertical introduction, horizontal amplification, environmental persistence, and therefore symptomatic shedding collectively sustain viral maintenance. Understanding the relative contribution of these interconnected transmission routes, therefore, is essential for developing effective and sustainable control strategies.

### 4.1. Horizontal Transmission and Environmental Persistence

Horizontal transmission remains the principal mechanism responsible for the rapid within-flocks dissemination. The fecal-oral route is considered the dominant pathway of environmental spread, particularly under intensive production conditions where large quantities of fecal material accumulate within confined housing systems. Infected birds begin shedding virus shortly after infection, resulting in the continuous contamination of litter, feed, drinking water systems, ventilation surfaces, and farm equipment [16,72].

A key determinant of horizontal transmission is the remarkable environmental stability of ARV. As a non-enveloped virus, ARV exhibits substantially greater resistance to environmental inactivation than many enveloped avian respiratory viruses. Experimental studies have demonstrated prolonged viral survival in water, litter, feathers, and feed materials, indicating that environmental reservoirs likely contribute significantly to viral persistence between flock cycles [73]. Recent industry-based reviews further emphasized that ARVs display considerable tolerance to commonly used disinfectants, thereby complicating environmental decontamination in commercial production systems [15,53].

Although enteric transmission predominates, respiratory and aerosol-associated exposure may also contribute to viral dissemination under field conditions. Infectious virus has been isolated from the respiratory tract of experimentally infected chickens, and intranasal inoculation reliably establishes infection [74]. Dust-rich environments including hatcheries, transport systems, layer houses, and live-bird markets, may therefore, facilitate short-distance aerosol-associated transmission, particularly under conditions of poor ventilation and high stocking density. Nevertheless, the quantitative contribution of airborne transmission to ARV epidemiology remains poorly defined and warrants further investigation.

Subclinical infection further complicates transmission control. Birds showing no overt clinical signs may continue shedding virus and sustain environmental contamination while remaining undetected within commercial flocks. This inapparent shedding likely contributes substantially to the frequent re-emergence of ARV following routine cleaning and disinfection procedures. Mechanical transmission through damaged skin may represent an additional, although less extensively studied, route of viral entry. Commercial broiler production systems commonly predispose birds to footpad dermatitis, skin abrasions, and minor skin trauma, which may facilitate viral invasion under conditions of high environmental viral pressure [75]. The hatchery represents a particularly important epidemiological interface linking vertical and horizontal transmission. Eggs carrying vertically transmitted virus may contaminate incubators, chick-handling equipment, transport containers, hatchery dust, and ventilation systems. Experimental observations demonstrated that ARV-positive chicks efficiently transmitted virus to contact birds during the first weeks post-hatch [76].

**Figure 3:**
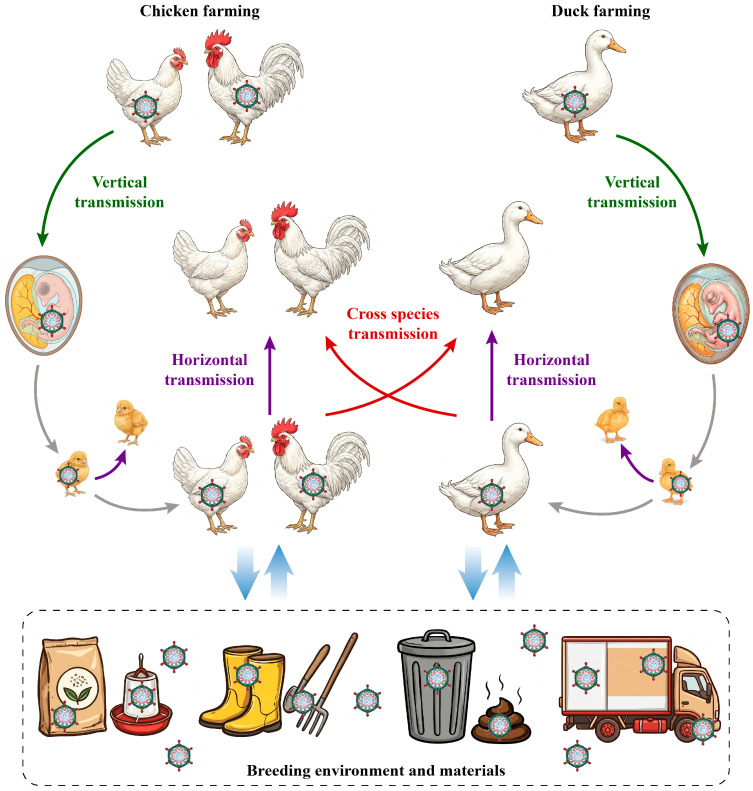
Integrated transmission ecology of avian orthoreoviruses (ARVs) in chicken and duck production systems. Conceptual overview of ARV transmission pathways in commercial poultry production. Vertical transmission (green arrows) enables dissemination from infected breeder flocks to progeny through eggs, introducing infected hatchlings into production systems. Horizontal transmission (purple arrows) occurs via direct contact, fecal-oral exposure, and contaminated environmental sources, including litter, feed, water, equipment, transport vehicles, hatcheries, and other fomites. Cross-species transmission at the chicken–waterfowl interface (red arrows) may facilitate viral exchange, host adaptation, and genomic reassortment. Environmental reservoirs contribute to viral persistence between production cycles because ARVs are environmentally stable non-enveloped viruses. Subclinically infected birds can continuously shed virus and serve as important sources of viral maintenance and dissemination within poultry production networks. Gray arrows indicate the growth route of chickens or ducks. Gemini 3.5 (Google, Mountain View, CA, USA) was used exclusively to generate basic graphic elements, including schematic illustrations of chickens, ducks, eggs, viruses, and farming-related materials. Each generated element was subjected to rigorous manual inspection to rule out any potential scientific inaccuracies. All elements were subsequently imported into Adobe Illustrator (version 2025), where the authors performed comprehensive manual integration, typesetting, and visual optimization.

Modern poultry production systems likely amplify these transmission dynamics substantially. Short intervals between flock placements frequently limit effective environmental decontamination, allowing residual virus to persist on housing surfaces, water lines, ventilation ducts, and transport equipment. Accordingly, horizontal transmission extends beyond direct bird-to-bird spread and should be considered an environmentally sustained process reinforced by persistent contamination, hatchery-mediated dissemination, and continuous flock replacement. The comparative schematics in Figure 3 summarize the distinct transmission pathways, environmental reservoirs, and farming system characteristics that drive ARV persistence in intensive chicken versus semi-intensive duck production systems.

### 4.2. Vertical Transmission and Early Life Amplification

Vertical transmission has long been recognized as an important contributor to ARV persistence, although its relative epidemiological importance has historically remained a matter of debate. Early experimental studies demonstrated that infected breeder birds could transmit the virus to progeny through eggs, thereby establishing infection before hatching [77,78]. Subsequent investigations revealed that vertical transmission efficiency varies considerably among viral strains. Certain arthrotropic strains exhibited substantially higher virus recovery rates from embryos and hatchlings than other isolates, suggesting that reproductive-tract tropism and viral replication kinetics may influence the success of vertical transmission [79].

Recent surveillance studies conducted in China provide increasing evidence that vertical transmission contributes to the ongoing field circulation of ARV. Hatchery investigations identified viral RNA and infectious virus in dead embryos, weak chicks, and newly hatched progeny originating from infected breeder flocks [76]. Importantly, emerging evidence suggests that vertical transmission may involve both hens and roosters, thereby broadening the traditional epidemiological framework of breeder-associated ARV transmission (Figure 3). Vertically infected chicks are epidemiologically important not only because they harbor the virus at hatch but also because they serve as efficient early-life amplifiers for subsequent horizontal spread. Experimental studies demonstrated rapid viral dissemination from infected chicks to contact birds during the first weeks of life, accompanied by severe tendon lesions and sustained viral shedding [76]. Collectively, these observations suggest that vertical transmission functions less as an isolated reproductive event than as a continuous reseeding mechanism that introduces infected progeny into commercial production systems before post-hatch biosecurity measures can be effectively implemented. Once introduced into hatcheries or broiler houses, subsequent horizontal dissemination rapidly amplifies viral circulation throughout the flock.

Vertical transmission has also been documented in waterfowl-origin orthoreoviruses. Experimental infection studies involving novel duck reovirus (NDRV) have detected viral RNA or infectious virus in embryos and newly hatched ducklings, together with reproductive-tract lesions in infected breeder ducks [80]. Similar to observations in chickens, some infected ducklings may initially appear clinically normal but subsequently develop systemic lesions later in life (Figure 3).

### 4.3. Waterfowl-Origin Reoviruses and Expanding Transmission Ecology

The emergence of novel duck reovirus (NDRV) and goose-origin reovirus (GRV) has substantially increased the epidemiological complexity of avian orthoreoviruses in China. Unlike classical chicken-origin ARVs, many waterfowl-origin strains exhibit broader tissue tropism and are frequently associated with systemic disease, including hepatic necrosis, splenic lesions, immunosuppression, and elevated mortality [81,82]. The rapid expansion of integrated poultry production systems has increased ecological contact among chickens, ducks, geese, backyard poultry, and live-market populations. Shared water systems, live-poultry markets, transport vehicles, and mixed-species farming environments may therefore create permissive interfaces for occasional interspecies transmission and genomic reassortment. Whole-genome analyses revealed that several waterfowl-origin orthoreoviruses possess reassortant genomic constellations containing segments closely related to chicken-origin strains [30,52]. These findings suggest that waterfowl may serve as epidemiologically relevant reservoirs or intermediary hosts. Recent reviews further emphasized the importance of systematic surveillance at the domestic waterfowl–chicken interface, because continued viral exchange within these ecological interfaces may facilitate the emergence of reassortant variants with altered host adaptability, tissue tropism, and pathogenicity [83].

Of particular concern, certain duck-origin reassortant strains have demonstrated enhanced pathogenicity in experimentally infected chickens [30,71]. Experimental infection studies showed that highly pathogenic duck reoviruses induced severe focal necrosis and hemorrhagic lesions in the liver, spleen, bursa of Fabricius, and thymus of inoculated birds, confirming that chickens can serve as susceptible hosts for selected duck-origin reoviruses. Although the long-term epidemiological stability and field transmissibility of such host-adapted or host-permissive reassortants remain uncertain, these observations highlight the increasingly interconnected evolutionary landscape linking chicken- and waterfowl-origin orthoreoviruses.

The expanding host ecology of ARV has important implications for disease surveillance. Surveillance systems focused exclusively on chickens are unlikely to capture the full diversity of circulating orthoreoviruses. Integrated multispecies surveillance involving chickens, ducks, geese, hatcheries, environmental samples, and live-market interfaces will likely play an increasingly important role in the early detection of emerging reassortant variants. From an ecological perspective, waterfowl-associated orthoreoviruses may constitute an important reservoir or source of genetic diversity, capable of contributing novel genome segments to circulating chicken-origin ARV populations.

### 4.4. Synthesis: Integrated Transmission Dynamics and Control Implications

Collectively, current evidence supports an integrated transmission model in which vertical transmission continuously introduces infected progeny into hatchery and broiler production, whereas horizontal transmission subsequently amplifies viral dissemination within flocks and production environments. The interaction between these transmission pathways likely explains the remarkable persistence of ARV despite widespread vaccination and routine biosecurity implementation. Figure 3 presents an integrated transmission model in which breeder-associated vertical introduction continuously seeds hatcheries and broiler houses, horizontal amplification drives rapid within-flock dissemination, and the waterfowl–chicken interface acts as an ecological bridge for cross-species transmission and reassortment. Waterfowl-associated orthoreoviruses further increase epidemiological complexity by serving as potential reservoirs or source of reassortant and antigenically divergent variants. Consequently, control strategies focused exclusively on broiler-stage disease are unlikely to achieve durable suppression of viral circulation.

Effective long-term control will likely require coordinated interventions across multiple stages of the poultry production chain, including breeder surveillance, hatchery sanitation, environmental decontamination, early-life molecular diagnostics, the optimization of vaccination schedules and vaccine deployment strategies, and integrated multispecies surveillance. Furthermore, persistent transmission through vertically infected progeny and environmentally contaminated production systems may create conditions that favor prolonged viral circulation, repeated exposure, and sustained host–virus interactions, all of which are highly relevant to the immunomodulatory consequences discussed in the following section.

## 5. Immune Interference and Pathogen Interaction Networks

A major conceptual advance in understanding ARV pathogenesis is the recognition that immune dysregulation represents a central determinant of disease outcome rather than merely a secondary consequence of tissue damage. Increasing experimental and field evidence demonstrates that ARV actively modulates both innate and adaptive immune responses, thereby promoting viral persistence, increasing susceptibility to secondary pathogens, and potentially compromising vaccine performance under commercial production conditions.

Within intensive poultry systems, where multiple viral and bacterial agents frequently co-circulate, even transient impairment of immune competence may substantially alter flock-level disease dynamics, secondary infection risk, and production performance. Consequently, ARV should no longer be viewed solely as an arthrotropic pathogen associated with viral arthritis and tenosynovitis, but rather as a systemic immunomodulatory virus capable of influencing polymicrobial disease ecology in modern poultry production systems.

### 5.1. Molecular Basis of ARV-Induced Immunosuppression

ARV infection affects multiple lymphoid organs, including the bursa of Fabricius, thymus, spleen, and gut-associated lymphoid tissues. Histopathological studies have consistently demonstrated lymphoid depletion, follicular atrophy, and the impaired development of immune organs, indicating a marked disruption of both humoral and cell-mediated immune functions [54]. Importantly, these alterations may persist beyond the acute phase of infection, potentially contributing to prolonged immune dysfunction and increased susceptibility to secondary infections under field conditions.

Beyond direct lymphoid injury, ARV encodes several proteins capable of antagonizing host antiviral signaling pathways. A recent review summarized immune-evasion strategies employed by ARV, particularly highlighting the roles of σA and p17 proteins in suppressing innate immune activation [84]. σA exhibits strong double-stranded RNA-binding activity, enabling the sequestration of viral replication intermediates and limiting the activation of intracellular RNA sensors, including the MDA5- and PKR-associated signaling pathways [31]. Through the inhibition of early interferon induction, ARV can effectively weaken the establishment of an intracellular antiviral state during the early phase of infection.

The non-structural protein p17 also acts as an important mediator of immune evasion. Recent studies have demonstrated that p17 interacts with components of the STING-associated signaling axis, including IFI16 and PQBP1, thereby attenuating downstream interferon-regulatory signaling [32,33]. Collectively, these findings indicate that ARV immune evasion involves the coordinated disruption of multiple innate immune checkpoints rather than the inhibition of a single pathway.

Additional evidence from waterfowl-origin orthoreoviruses further supports this concept. A recent characterization of goose-origin ARV strain SD0407 demonstrated efficient replication in goose embryo fibroblasts without detectable induction of type I, type II, or type III interferon responses [65]. Transcriptomic analysis further revealed only weak activation of innate immune signaling pathways together with the downregulation of metabolic and cytoskeleton-associated genes, suggesting that some waterfowl-origin strains may possess highly effective interferon-suppressive mechanisms.

Collectively, these observations support the concept that ARV-associated immunosuppression may represent an evolutionarily advantageous viral strategy that promotes the persistence, transmission, and ecological success of the virus. Together, immune evasion and immunomodulation may help sustain genetically diverse ARV populations under intensive production conditions.

### 5.2. ARV as a Driver of Polymicrobial Disease Complexity

The immunosuppressive effects of ARV can profoundly reshape the microbial ecology of commercial poultry flocks. Field investigations frequently identify co-infections involving ARV and pathogens such as Fowl adenovirus serotype 4, Infectious bursal disease virus, Chicken anemia virus, *Staphylococcus aureus*, *Mycoplasma synoviae*, *Riemerella anatipestifer*, and pathogenic *Escherichia coli* [34,35,85]. Recent studies involving novel duck reovirus (NDRV) have further expanded our understanding of orthoreovirus-associated immunopathology. Severe NDRV infection induces extensive splenic necrosis and marked depletion of splenic macrophages, leading to immunosuppression, increased susceptibility to secondary infections, and impaired vaccine responsiveness [81]. These findings provide mechanistic insight into how waterfowl-origin orthoreoviruses disrupt systemic immune homeostasis and promote polymicrobial disease progression.

Experimental co-infection models consistently demonstrate aggravated disease severity compared with single-pathogen infection. Chickens simultaneously infected with ARV and *S. aureus* developed more severe tenosynovitis, higher bacterial loads in synovial tissues, and increased mortality [35]. Similarly, co-infection involving ARV and FAdV-4 enhanced viral replication, intensified tissue damage, and dysregulated inflammatory cytokine responses [34]. These findings support the concept that ARV functions as an important facilitating pathogen that lowers the threshold for opportunistic pathogens to establish systemic infection. By impairing innate and adaptive immune competence, ARV may amplify polymicrobial disease complexity and destabilize flock health under intensive production conditions. Particularly concerning is the impact of ARV on vaccine-mediated protection against unrelated pathogens. A recent study demonstrated that chickens infected with ARV prior to FAdV-4 vaccination developed significantly weaker antibody responses and only partial protection following a virulent challenge [86].

### 5.3. ARV-Mediated Interference with Vaccination Programs

The immunomodulatory properties of ARV have important implications for vaccination strategies in modern poultry production. Commercial broiler flocks are commonly exposed to multiple live and inactivated vaccines during the early post-hatch period, and immunological interactions among these vaccine exposures may significantly influence vaccine efficacy and immune development. ARV-induced immune dysregulation may further impair the development of protective responses to unrelated vaccines. Recent experimental studies have demonstrated that concurrent administration of live ARV and IBDV vaccines can reduce ARV-specific antibody responses and weaken protection against subsequent virulent ARV challenge [87]. These findings indicate that vaccine-associated immune interference may emerge when multiple immunologically demanding vaccines are administered during the early phase of immune maturation. Field observations further suggest that neonatal vaccination with certain classical live ARV vaccine strains may negatively affect gastrointestinal function, resulting in reduced feed conversion efficiency and impaired body weight gain in broiler chicks [88]. Moreover, maternally derived antibodies from S1133-vaccinated breeder hens were insufficient to fully prevent infection by homologous live ARV vaccine viruses in progeny birds, highlighting the limitations of classical vaccine protection under contemporary production conditions [5].

Several biological mechanisms may contribute to these observations, including transient bursal impairment induced by live IBDV vaccines and ARV-associated suppression of lymphocyte proliferation and immune responsiveness. Under intensive vaccination schedules, the cumulative immunological burden generated by repeated antigen exposure and concurrent viral replication may exceed the functional capacity of the immature immune system in young chicks. These findings emphasize the importance of optimizing vaccination timing, antigen compatibility, and flock immune status when designing field immunization programs. Wider spacing between live vaccines, enhanced surveillance for immunosuppressive pathogens, and greater use of non-replicating or vectored vaccine platforms may help reduce immune interference under intensive production conditions.

### 5.4. Cytokine Dysregulation and Systemic Production Consequences

ARV infection is associated with pronounced dysregulation of inflammatory cytokine responses. Infected tissues frequently exhibit elevated expression of IL-1β, IL-6, TNF-α, and IFN-γ, which is closely associated with the severity of synovial inflammation and tendon injury [35,54,89]. Sustained inflammatory activation promotes mononuclear cell infiltration and contributes to tissue fibrosis, locomotor impairment, and chronic losses in production performance. Simultaneously, the suppression of antiviral interferon signaling may impair efficient viral clearance and permit prolonged viral persistence within infected tissues. The coexistence of excessive inflammatory activation and incomplete antiviral control likely represents a major immunopathological basis for the persistent decline in flock performance following ARV outbreaks.

Importantly, the consequences of ARV-associated immune dysfunction extend beyond overt clinical disease. Subclinically infected flocks frequently exhibit impaired growth performance, reduced flock uniformity, decreased feed conversion efficiency, and increased antimicrobial usage associated with secondary bacterial infections [5,90]. Because many of these effects develop gradually and remain partly inapparent at the flock level, the true economic burden of ARV is likely underestimated by surveillance systems focused primarily on mortality and severe lameness outbreaks. These findings reinforce the concept that ARV should be regarded not only as an infectious agent but also as a chronic productivity-limiting pathogen capable of reshaping long-term flock health and production efficiency.

### 5.5. Synthesis: An Integrated Perspective on ARV Immune Interference

Collectively, current evidence indicates that ARV exerts broad immunomodulatory effects that extend well beyond localized tendon and joint pathology. Through the coordinated disruption of innate antiviral signaling, lymphoid integrity, inflammatory regulation, and vaccine responsiveness, ARV establishes conditions favorable for prolonged viral persistence and the expansion of secondary pathogens.

The interaction among ARV-associated immunosuppression, polymicrobial infection, and vaccine interference likely represents one of the major drivers of disease persistence in intensive poultry production systems. Rather than functioning as isolated biological events, these processes appear to mutually reinforce one another and progressively destabilize flock immune homeostasis. This integrated immunopathological perspective has important implications not only for disease pathogenesis but also for commercial poultry production. Even subclinical ARV-associated immune dysfunction may impair vaccine responsiveness, increase susceptibility to secondary bacterial and viral infections, reduce flock uniformity, compromise growth performance, increase feed conversion ratios, prolong time to market weight, and elevate antimicrobial usage. Because these consequences often develop gradually and remain difficult to quantify under field conditions, their cumulative economic impact may exceed losses directly attributable to viral arthritis, tenosynovitis, or mortality.

Effective long-term control strategies will therefore require the simultaneous optimization of early ARV diagnosis, vaccination schedules, environmental biosecurity, and the prevention of concurrent immunosuppressive infections. Evaluation of ARV control programs should increasingly incorporate production performance indicators, including growth rate, flock uniformity, vaccine responsiveness, and antimicrobial consumption, in addition to traditional clinical outcomes. In addition, viral proteins involved in interferon antagonism and immune modulation may represent promising targets for next-generation vaccine development and antiviral intervention.

Accordingly, future ARV research should increasingly consider the virus within a systems-biology framework that integrates virology, immunology, microbiome dynamics, and production ecology rather than investigating ARV infection as an isolated disease entity.

## 6. Integrated Prevention and Control Strategies

The rapid diversification of contemporary ARV populations in China has progressively undermined the effectiveness of conventional control strategies that rely predominantly on fixed vaccination programs. Continuous genotype turnover, genomic reassortment, antigenic drift, vertical transmission, and the widespread co-circulation of genetically heterogeneous strains collectively pose a major challenge to the long-term efficacy of classical vaccines and conventional flock-level interventions.

Under modern intensive poultry production systems, ARV control can no longer be limited to the prevention of clinical arthritis or tenosynovitis alone. Effective control increasingly depends on reducing viral circulation across the entire production pyramid, interrupting breeder-associated and hatchery-mediated transmission, minimizing opportunities for reassortment, and limiting environmental persistence across interconnected poultry production networks.

Accordingly, contemporary ARV control requires an integrated risk-management framework that encompasses genomic surveillance, adaptive vaccination, biosecurity, breeder management, and flock health optimization. Such a framework should be sufficiently flexible to respond to continuous viral evolution and regional differences in genotype prevalence.

### 6.1. Limitations of Classical Vaccines and Constraints of Autogenous Strategies

The declining efficacy of classical ARV vaccines is primarily attributable to the increasing antigenic divergence between historical vaccine strains and contemporary field isolates. Commercial attenuated vaccines currently used in China are largely derived from genotype cluster I strains, whereas most circulating Chinese field strains now cluster within genotypes II-VII [25,40]. Cross-neutralization and challenge studies consistently demonstrated markedly reduced protection against heterologous strains, particularly those carrying extensive amino acid variation within the σC globular head domain [91,92]. This growing antigenic mismatch has become a major factor contributing to vaccine-breakthrough outbreaks in commercial poultry flocks and highlights the limited breadth of protection conferred by historically derived vaccine strains. Consequently, vaccine efficacy can vary considerably across regions depending on the local genotype composition and epidemiological context. A recent systematic review further emphasized that currently available commercial vaccines fail to provide adequate protection against the expanding diversity of ARV variants worldwide. High mutation rates, extensive σC variability, and frequent reassortment collectively reduce vaccine matching efficiency and increasingly compromise routine immunization programs [24,53].

To compensate for insufficient cross-protection, the poultry industry has increasingly adopted autogenous inactivated vaccines tailored to locally circulating outbreak strains. These vaccines may provide useful short-term homologous protection during regional epidemics and can partially reduce clinical losses at the company level [93,94]. Nevertheless, several inherent limitations constrain the long-term sustainability of autogenous vaccination strategies. First, most autogenous vaccines provide relatively narrow antigenic coverage. In regions where multiple genotypes co-circulate simultaneously, monovalent or limited multivalent formulations are unlikely to provide sufficiently broad protection. Second, the production timeline of autogenous vaccines frequently lags behind the pace of ongoing viral evolution. Under conditions of rapid genotype turnover, vaccine strains may already have reduced epidemiological relevance by the time field deployment occurs.

An additional concern is the evolutionary pressure imposed by repeated application of narrowly targeted vaccines. Sustained immune selection may favor the persistence and expansion of antigenically divergent variants that are less effectively controlled by vaccine-induced immunity, thereby promoting genotype turnover and increasing population complexity [24,40]. This phenomenon resembles vaccine-associated lineage replacement observed in several other rapidly evolving RNA viruses [41].

Current reliance on inactivated autogenous vaccines also limits the induction of robust mucosal and cell-mediated immunity. Because most available formulations predominantly stimulate humoral immune responses, they may be insufficient to fully suppress viral replication at mucosal entry sites or within tendon-associated target tissues. The absence of live vaccines matched to contemporary genotypes further restricts development of optimized prime-boost immunization strategies capable of inducing broader and more durable protection. Consequently, autogenous vaccines should be regarded as transitional epidemiological tools rather than definitive long-term solutions. Their greatest value may lie in providing short-term outbreak control while broader-spectrum vaccine platforms continue to be developed and validated.

### 6.2. Next-Generation Vaccines and Adaptive Immunization Strategies

Long-term ARV control will likely require vaccine platforms capable of being updated more rapidly than conventional whole-virus vaccines. Future vaccine development should increasingly emphasize antigenic breadth, rapid adaptability, and compatibility with the evolving epidemiology of field strains rather than relying on static strain selection.

Recombinant viral-vector vaccines based on Marek’s disease virus (MDV) or Newcastle disease virus (NDV) expressing σC- or σB-associated antigens have demonstrated promising immunogenicity and partial cross-protective efficacy in experimental studies [95,96]. Importantly, σB has increasingly been recognized as an antigenically relevant structural protein involved in the induction of neutralizing responses, suggesting that future vaccine design may benefit from multivalent antigen combinations rather than reliance on σC alone. These vector platforms may support earlier and more uniform immunization, including compatibility with in ovo vaccination systems widely used in modern poultry production. Such early-life vaccination strategies could be particularly valuable for reducing the vulnerable post-hatch window during which maternally derived antibodies decline and horizontal transmission may accelerate.

Subunit and epitope-focused vaccines represent another promising direction for next-generation ARV vaccine development. Structural analyses of σC have enabled the identification of relatively conserved antigenic regions that may mediate broader cross-genotype protection [46,97]. However, whether conserved epitopes alone can provide sufficient field-level protection against highly heterogeneous ARV populations remains uncertain and requires further validation under commercial production conditions. A promising strategy may involve combining conserved protective epitopes with genotype-representative antigens to balance cross-protective breadth and strain-specific immunity.

Nucleic acid vaccines, including mRNA and circular RNA (circRNA) platforms, have recently attracted substantial attention because of their rapid updateability and scalable manufacturing potential. A circRNA-based vaccine encoding σC from the novel duck reovirus induced strong humoral immune responses and complete homologous protection in challenge experiments [98]. Although these platforms remain at an early stage of development for ARV and related avian orthoreoviruses, these findings highlight the potential utility of rapidly adaptable vaccine technologies for pathogens characterized by continuous antigenic evolution. The success of mRNA vaccines in human medicine has further stimulated interest in applying similar platform technologies to rapidly evolving veterinary pathogens, including ARV. A recent review on preventive immunology in livestock further summarized emerging technologies including mRNA vaccines, nanovaccines, monoclonal antibodies, bacteriophage-based strategies, probiotics, and CRISPR-assisted antiviral tools [99]. Although many of these technologies remain experimental, they may eventually support precision immunization strategies tailored to regional genotype distributions and evolving epidemiological risks.

Optimization of vaccination schedules is equally important. Recent studies have demonstrated that closely timed administration of live ARV and infectious bursal disease virus (IBDV) vaccines can reduce vaccine responsiveness because of immunological interference between live vaccine viruses [87]. Accordingly, adequate spacing between live immunizations may be necessary to preserve immune responsiveness in immunologically immature broiler chicks, particularly under intensive production systems characterized by dense early-life vaccination schedules and concurrent exposure to immunosuppressive pathogens. The comparative advantages, limitations, current applications, and future prospects of classical and next-generation ARV vaccine platforms are summarized in Table 1. Collectively, these advances support a gradual transition from static, genotype-restricted formulations toward adaptive immunization strategies informed by genomic surveillance and evolving field epidemiology.

### 6.3. Genomic Surveillance and Precision Epidemiology

Although whole-genome sequencing has substantially improved our understanding of ARV evolution, reassortment, and transmission dynamics, important limitations remain within current surveillance systems. First, surveillance activities are frequently concentrated in clinically affected flocks, creating potential sampling bias and limiting the detection of subclinical infections. Second, many diagnostic investigations continue to rely primarily on partial σC sequencing, which may underestimate genome-wide diversity and fail to identify reassortment events. Third, surveillance coverage remains uneven across geographic regions, poultry sectors, and host species, with waterfowl, hatcheries, environmental reservoirs, and live-bird marketing systems often being underrepresented. Fourth, genomic data are rarely integrated with epidemiological, production performance, and vaccination history datasets, restricting comprehensive risk assessment and outbreak prediction.

These limitations collectively hinder the accurate evaluation of ARV evolutionary dynamics and transmission networks. Future surveillance frameworks should therefore prioritize routine whole-genome sequencing, standardized metadata collection, real-time data sharing, and coordinated multispecies monitoring. Integration of genomic epidemiology with production and health management data may ultimately facilitate precision surveillance and evidence-based control strategies for emerging ARV variants.

### 6.4. Biosecurity, Flock Health, and Integrated Control Frameworks

Vaccination alone is unlikely to achieve sustainable control of ARV without comprehensive biosecurity and flock health management. Because ARV is environmentally stable and relatively resistant to inactivation, rigorous sanitation remains essential across breeder farms, hatcheries, transportation systems, and broiler operations [15]. Core biosecurity measures include all-in/all-out flock management, strict hatchery hygiene, effective cleaning and disinfection protocols, the restriction of personnel and equipment movement, and the reduction of environmental contamination in housing and water systems. A practical industry guideline emphasized that breeder immunization represents one of the most critical interventions for controlling reovirus-associated arthritis and tenosynovitis because it can simultaneously reduce vertical transmission and enhance maternally derived antibody protection in progeny chicks [101]. Particular attention should be directed toward hatcheries, which likely function as major epidemiological amplification hubs linking vertical and horizontal transmission. ARV-contaminated eggs may disseminate the virus throughout incubators, chick-processing systems, transport equipment, and hatchery dust, thereby facilitating rapid flock-wide spread shortly after hatch.

Environmental and nutritional management may also influence disease outcomes and flock resilience. Adequate ventilation, optimized stocking density, improved litter quality, water sanitation, and reduced mycotoxin exposure can collectively reduce physiological stress and help maintain immune competence. Nutritional supplementation with selenium, antioxidant vitamins, probiotics, and selected phytogenic compounds has been proposed as a supportive strategy capable of partially mitigating subclinical immunosuppression [53]. Nevertheless, robust field evidence supporting the consistent efficacy of many immunomodulatory additives remains limited. Breeder surveillance remains central to reducing vertical transmission. Because breeder flocks constitute a major source of vertically infected progeny, the early identification and management of infected breeders are likely to generate benefits across the entire production pyramid. Monitoring breeder flocks through routine molecular diagnostics, the removal or management of persistent shedders, and the strengthening of hatchery sanitation protocols may substantially reduce the introduction of infected progeny into commercial production systems.

Increasingly, genomic surveillance is becoming an indispensable component of ARV control programs. Routine implementation of whole-genome sequencing can provide an early warning for emerging reassortant variants, facilitate vaccine-strain selection, and improve the reconstruction of transmission pathways across production systems. Whole-genome sequencing combined with phylodynamic analysis can improve the detection of reassortant variants, monitor genotype replacement, and identify emerging transmission networks in real time [9,24]. Although artificial intelligence-assisted epidemiological prediction remains at an early stage in poultry medicine, the future integration of genomic, environmental, and production data may substantially improve outbreak forecasting and precision risk assessment.

Overall, sustainable ARV control will likely require the coordinated integration of vaccination, breeder management, genomic surveillance, environmental decontamination, hatchery biosecurity, and flock health optimization rather than reliance on any single intervention. Such a multifaceted approach offers the greatest potential for reducing both the clinical and economic burden of ARV infection and the evolutionary opportunities available to the virus within modern poultry systems.

## 7. Future Perspectives and Conclusions

The recent evolution of ARV in China illustrates how segmented RNA viruses can adapt rapidly under conditions of intensive animal production, sustained immunological pressure, and extensive host connectivity. During the past decade, ARV has evolved from a relatively localized cause of viral arthritis into a genetically diverse and epidemiologically complex pathogen associated with vaccine-breakthrough infection, multispecies circulation, reassortment, immunomodulation, and chronic productivity loss. A recent global systematic review confirmed that ARVs exhibit extensive geographical heterogeneity and substantial genotypic diversity, with at least six major phylogenetic groups currently recognized worldwide [15,53]. Notably, the evolutionary trends observed in China closely mirror those reported in North America, Europe, and other poultry-producing regions, suggesting that ARV diversification is a global evolutionary phenomenon rather than a region-specific event. Frequent reassortment and ongoing antigenic diversification increasingly complicate vaccine matching and underscore the need for continuous adaptation of immunization strategies.

Future ARV research should increasingly move beyond descriptive genotype classification toward integrated investigations of viral evolution, host interactions, immune modulation, and transmission ecology. Particular emphasis should be placed on linking genomic variation with phenotypic outcomes, including pathogenicity, tissue tropism, transmissibility, antigenicity, and vaccine responsiveness. Such genotype-to-phenotype integration remains one of the most important unresolved challenges in ARV biology. Whole-genome sequencing integrated with temporal, geographic, and host-associated epidemiological metadata will likely become indispensable for monitoring reassortment dynamics, predicting genotype emergence, and reconstructing transmission networks [24]. At the same time, advances in molecular diagnostics are expected to substantially improve surveillance sensitivity and outbreak response. An updated review of waterfowl reovirus infections emphasized the growing importance of high-throughput molecular diagnostics and genome-based detection technologies for the rapid identification of emerging variants [83]. The integration of such diagnostic platforms with genomic epidemiology and real-time surveillance systems may form the foundation of next-generation ARV monitoring programs. Structural vaccinology and rapidly updateable vaccine platforms, including mRNA, circRNA, and vector-based systems-may substantially improve the ability to respond to ongoing antigenic diversification. Nevertheless, successful implementation will depend not only on technological innovation but also on manufacturing scalability, regulatory approval, economic feasibility, and practical applicability within commercial poultry production systems [14].

Another emerging concern involves the increasingly complex ecological interface among chickens, ducks, geese, and potentially wild avian hosts. Continued surveillance across these interconnected host populations has been repeatedly emphasized as critical for the early identification of reassortant variants with altered pathogenicity or host range [30,52]. The recent characterization of a goose-origin ARV strain exhibiting potent interferon-suppressive activity [65] further raises concern that waterfowl populations may serve as reservoirs or sources of immune-evasive variants with spillover potential into commercial poultry systems. These findings reinforce the need for a multispecies surveillance framework encompassing domestic poultry, waterfowl, wild birds, and environmental reservoirs, thereby aligning ARV monitoring with broader One Health principles. Accordingly, future surveillance should move from isolated host-specific programs toward unified multispecies monitoring frameworks integrating domestic poultry, waterfowl, hatcheries, environmental reservoirs, and the wild-bird interfaces [102].

In conclusion, ARV has evolved into a genetically diverse and epidemiologically complex poultry pathogen whose persistence is driven by the interplay of genomic evolution, transmission ecology, immune modulation, and intensive production practices. Continued diversification through mutation, reassortment, and interspecies transmission poses substantial challenges to vaccine efficacy and disease control. Future success will depend on the integration of whole-genome surveillance, adaptive vaccination, biosecurity enhancement, and multispecies epidemiological monitoring within a unified control framework. Beyond its significance to poultry health, ARV provides an informative model for understanding how rapidly evolving segmented RNA viruses adapt, persist, and emerge within intensive agricultural ecosystems, offering broader insights into the prevention and management of emerging viral diseases in animal production systems.

## Figures and Tables

**Table 1:** Comparative advantages, limitations, and future prospects of current and emerging vaccine strategies for ARV control.

Vaccine Strategy	Advantages	Limitations	Current Application	Future Potential	References
Classical live-attenuated and inactivated vaccines (such as S1133, 1733, and 2408 strains)	Established manufacturing infrastructure, relatively low production cost, and proven ability to induce protective humoral immunity against homologous or closely related strains.	Reduced antigenic compatibility with many contemporary field isolates belonging to genotypes GII-GVII, resulting in variable cross-protective efficacy and incomplete suppression of viral transmission.	Remain the predominant vaccine platforms used in breeder and layer flocks worldwide; however, vaccine-breakthrough infections are increasingly reported in regions with high ARV genetic diversity.	Likely to retain value as baseline immunization tools within heterologous prime-boost or multicomponent vaccination programs incorporating updated antigens.	[5,40,53]
Autogenous inactivated vaccines	Allow rapid incorporation of locally circulating strains and often provide improved homologous protection during regional outbreaks.	Limited antigenic breadth, delayed production relative to ongoing viral evolution, and potential inability to provide sustained protection against newly emerging variants.	Increasingly utilized by integrated poultry companies in China, the United States, and Israel as outbreak-management tools.	Future optimization may involve integration with real-time genomic surveillance and incorporation into multivalent or strain-updated vaccine formulations.	[24,93]
Recombinant viral-vectored vaccines (rMDV, rNDV expressing σC and/or σB)	Capable of delivering multiple protective antigens, compatible with in ovo vaccination, and potentially provide simultaneous protection against both ARV and vector-associated diseases.	Protective efficacy may be influenced by pre-existing vector immunity, transgene stability, expression efficiency, and regulatory considerations.	Currently confined to experimental and pre-commercial evaluation.	Represent promising next-generation platforms for rapid antigen updating and early-life immunization in intensive poultry production systems.	[95,96]
Subunit vaccines (baculovirus-, yeast-, or *E. coli*-expressed σC, σB, or epitope antigens)	Excellent biosafety profile, defined antigen composition, compatibility with differentiating infected from vaccinated animal (DIVA) strategies, and flexibility for rational antigen design.	Often require potent adjuvants, repeated administration, and optimization to overcome relatively limited cellular and mucosal immune responses.	Demonstrated encouraging protection in experimental challenge studies but have not yet achieved widespread commercial deployment.	Multivalent, chimeric, or structure-guided antigen designs incorporating conserved epitopes may improve cross-genotype protection.	[5,27,97]
Nucleic acid vaccines (mRNA and circRNA)	Rapid design and manufacturing, sequence adaptability, and ability to induce both humoral and cellular immune responses. CircRNA platforms additionally offer enhanced molecular stability.	Challenges include delivery efficiency, production cost, cold-chain requirements, and limited experience in large-scale poultry vaccination programs.	Currently at the proof-of-concept stage; recent studies demonstrated robust protection against homologous NDRV challenge in ducks.	May become highly adaptable vaccine platforms for responding to rapidly evolving ARV populations, particularly when combined with genomic surveillance and precision-vaccination strategies.	[98,99,100]

## Data Availability

All data are available within the manuscript.
